# Supplementary material for: Dosage individualization proposed for anti-gout medications among the patients with gout
Source: PLoS One. 2021 Sep 17;16(9):e0257082. doi: 10.1371/journal.pone.0257082 (PMC8448378; doi:10.1371/journal.pone.0257082)
Supplement: S1 Table — (DOCX) [file pone.0257082.s001.docx]

**S1 Table: Comorbidities of the patients** [23]

| **Comorbid conditions** | **ICD-11 classification*** | **Frequency (%)** |
| --- | --- | --- |
| Allergic reactions | 4A8Z | 1 (0.3) |
| Asthma | CA23 | 2 (0.5) |
| DM-2 | 5A11 | 7 (1.8) |
| Gastritis | DA42 | 1 (0.3) |
| Hyperlipidemia | 5C80.Z | 1 (0.3) |
| Hypertension | BA00 | 30 (7.8) |
| Hypertension, DM-2 | BA00, 5A11 | 9 (2.3) |
| Hypertension, DM-2, Hyperlipidemia | BA00, 5A11, 5C80.Z | 9 (2.3) |
| Hypertension, DM-2, Hyperthyroidism | BA00, 5A11, 5A02.Z | 3 (0.8) |
| Hypertension, Gastritis | BA00, DA42 | 1 (0.3) |
| Hypertension, Hyperthyroidism | BA00, 5A02.Z | 1 (0.3) |
| Hypertension, Renal impairment | BA00, GB6Z | 2 (0.5) |
| Hyperthyroidism | 5A02.Z | 37 (9.6) |
| Hyperthyroidism, DM-2 | 5A02.Z, 5A11 | 2 (0.5) |
| Hyperthyroidism, Hyperlipidemia | 5A02.Z, 5C80.Z | 1 (0.3) |
| Hyperthyroidism, Vitamin deficency | 5A02.Z, 5B55 | 1 (0.3) |
| Hypotension | BA20 | 2 (0.5) |
| Migraine | 8A80 | 11 (2.9) |
| No comorbid condition | QA02 | 260 (67.7) |
| Psychiatric disorder, Hyperthyroidism | 6E8Z, 5A02.Z | 1 (0.3) |
| Renal impairment | GB6Z | 2 (0.5) |
| Total |  | 384 (100) |
| ICD: International Statistical Classification of Diseases and Related Health Problems  *WHO Guidelines for ATC classification and DDD assignment 2020 23^rd^ edition | | |
