# Supplementary material for: Dosage individualization proposed for anti-gout medications among the patients with gout
Source: PLoS One. 2021 Sep 17;16(9):e0257082. doi: 10.1371/journal.pone.0257082 (PMC8448378; doi:10.1371/journal.pone.0257082)
Supplement: S2 Table — (DOCX) [file pone.0257082.s002.docx]

**S2 Table: Therapeutic category-wise medication use**

| **SN** | **Therapeutic category** | **Medicines** | **Used** | **No dose adjustment required (n,%)** | **Dose adjustment required** |
| --- | --- | --- | --- | --- | --- |
| 1 | NSAIDs | Aceclofenac 100 mg | 117 (30.5) | 117 (30.5) | - |
|  |  | Etoricoxib 60 mg | 1 (0.3) | 1 (0.3) | - |
|  |  | Etoricoxib 90 mg | 92 (24) | 85 (22.1) | 7 (1.8) |
|  |  | Etoricoxib 90/60 mg | 1 (0.3) | 1 (0.3) | - |
|  |  | Indometacin 25 mg | 1 (0.3) | 1 (0.3) | - |
|  |  | Indometacin 50 mg | 6 (1.6) | 6 (1.6) | - |
|  |  | Naproxen 250 mg | 2 (0.5) | 2 (0.5) | - |
|  | Total |  | 220 (57.5) | 213 (55.6) | 7 (1.8) |
| 2 | XOIs | Allopurinol 100 mg | 1 (0.3) | 1 (0.3) | - |
|  |  | Febuxostat 40 mg | 53 (13.8) | 49 (12.8) | 4 (1) |
|  |  | Febuxostat 80 mg | 7 (1.8) | 6 (1.6) | 1(0.3) |
|  | Total |  | 61 (15.9) | 56 (14.7) | 5 (1.3) |
| 3 | Alkaloids | Colchicine 500 mcg BD | 40 (10.4) | 36 (9.4) | 4 (1) |
| 4 | Glucocorticoids (Intermediate acting) | Methylprednisolone inj. 80 mg | 29 (7.6) | 28 (7.3) | 1(0.3) |
|  |  | Prednisolone 2.5 mg | 32 (8.3) | 29 (7.6) | 3(0.8) |
|  |  | Prednisolone 5 mg | 30 (7.8) | 27 (7) | 3(0.8) |
|  |  | Prednisolone 10 mg | 12 (3.1) | 10 (2.6) | 2(0.5) |
|  |  | Prednisolone 15 mg | 5 (1.3) | 3 (0.8) | 2(0.5) |
|  |  | Prednisolone 20 mg | 10 (2.6) | 9 (2.3) | 1(0.3) |
|  |  | Prednisolone 25 mg | 2 (0.5) | 2 (0.5) | - |
|  |  | Prednisolone 30 mg | 15 (3.9) | 14 (3.6) | 1(0.3) |
|  |  | Prednisolone 30/20/10 | 1 (0.3) | 1 (0.3) | - |
|  |  | Prednisolone 20/15/10 | 1 (0.3) | 1 (0.3) | - |
|  |  | Prednisolone 15/10/5/2.5 mg | 1 (0.3) | 1 (0.3) | - |
|  |  | Prednisolone 15/10/5 | 1 (0.3) | 1 (0.3) | - |
|  |  | Prednisolone 5/2.5 | 1 (0.3) | - | 1(0.3) |
|  |  | Triamcinolone 10 mg inj. | 1 (0.3) | 1 (0.3) | - |
|  | Total |  | 141 (36.9) | 127 (33.2) | 14 (3.8) |
| 5 | Atypical opioid | Tapentadol 50 mg | 3 (0.8) | 3 (0.8) | - |
| DHFRase: Dihydrofolate reductase; XOI: Xanthine oxidase inhibitor | | | | | |
