# Supplementary material for: Dosage individualization proposed for anti-gout medications among the patients with gout
Source: PLoS One. 2021 Sep 17;16(9):e0257082. doi: 10.1371/journal.pone.0257082 (PMC8448378; doi:10.1371/journal.pone.0257082)
Supplement: Attachment — Submitted filename: Dosage individualization proposed for anti-gout medications.docx [file pone.0257082.s003.docx]

| **Page & Line No.** | **Comments** |
| --- | --- |
| **Abstract** |  |
| Page 1 & line No. 23 | Kindly replace **unwillingness** with **willingness** in sentence |
| Page 2 & line No. 1- 2 | Kindly re-write this sentence, as meaning is not clear. |
| **Methods** |  |
| Page 4 & Line No. 5 and 7 | Kindly correct the spelling of “**radiological**” and “**first clinic**” |
| Page 5 & Line No. 21 | Kindly correct the typographical error, instead of “CDC guidance” write “CDC guidelines”. |
| Page 5 & Line No. 27 | Suggested to elaborate or outline the structure of self-developed tool used for data collection. |
| **Results** |  |
| Page 6 | Suggested to use either frequency or percentage to define the findings of variables in the manuscript. |
| **Discussion** |  |
| Page 7 & Line No. 27-28 | Kindly reform this sentence. |
| **Other** | Limitation of this study can critically affect the reproducibility of this study (non-adherence must be checked to maintain reproducibility) |
