# Supplementary material for: Dosage individualization proposed for anti-gout medications among the patients with gout
Source: PLoS One. 2021 Sep 17;16(9):e0257082. doi: 10.1371/journal.pone.0257082 (PMC8448378; doi:10.1371/journal.pone.0257082)
Supplement: Attachment — Submitted filename: Response to the reviewers and the editors comments_R3.docx [file pone.0257082.s004.docx]

**Response to reviewer's comments**

We are very much thankful to the editor and the reviewers for their valuable suggestions for our manuscript. We've tried our best to address all the concerns point-by-point and have also revised the manuscript in light of these, where necessary.

**Reviewer #2: All the comments have been satisfactorily addressed. No further comments. The manuscript can be accepted.**

**Response:** Thank you very much.

**Reviewer #3: All the comments have been address adequately by author. However, few more comments need to be address.**

**Comment 1 How the study will change the current prescribing pattern of anti-gout therapy with respect to dosage individualization (include in discussion) as the drugs were prescribed generally according to the treatment guidelines.**

**Response:** We've kept the following statement at the bottom of the Discussion section.

The study findings may help the practitioners select the optimum regimen of anti-gout therapy for the individual patient based on the dosage individualization proposed.

**Comment 2 In this study, patients diagnosed with gout were included, Kindly include the time period of therapy which they are taking like from one year or two year or naive patients (in tabular format in results).**

**Response:** We've indicated the duration of disease in tables 1 and 2, with clear units in years. The patients were on antigout therapy since they were diagnosed, but we followed the inclusion criteria (which we had already mentioned in our inclusion criteria) as:

Those patients who were freshly prescribed with antigout medications and/or those taking the same since last six months (from the date of data collection) were enrolled.

**Comment 3 Some of the patient were presented with their complications, was their therapy regimen was taken care of, like w.r.t drug interactions and other body parameters, if yes please include the status in results section.**

**Response:** The report was discussed with the prescribing physicians, and rest of the therapeutic decision making was left to them. The outcome was not followed up in the present research, as it was a cross-sectional study.

**Comment 4 On an average, how many follow-ups were taken w.r.t. each patient. Kindly include a rough average.**

**Response:** Since the study was cross-sectional, no follow up of the patient was performed within the study period. However, the prescribing physicians asked their patients to come up on follow up every six month.

**Comment 5 What was the dosage regimen of corticosteroids of patients with complications if it was tapered or escalated what pattern was used? Please elaborate.**

**Response:** Since there was no such report of complications with the dosage regimen of corticosteroids within the study period, this was not worrying case here.

**Other revisions performed:**

**Tables 1 and 2:** We've indicated the duration of disease in years, which was previously missing in table.

**Table 2:** We've shifted the descriptions of obesity status in the footnote to minimize the table length.
