# Supplementary material for: Dosage individualization proposed for anti-gout medications among the patients with gout
Source: PLoS One. 2021 Sep 17;16(9):e0257082. doi: 10.1371/journal.pone.0257082 (PMC8448378; doi:10.1371/journal.pone.0257082)
Supplement: Attachment — Submitted filename: PONE-D-20-38874.docx [file pone.0257082.s005.docx]

| **Page & Line No.** | **Comments** |
| --- | --- |
| **Abstract** |  |
| Page 2 & Line No.22 | As per submission guidelines, abbreviations of CKD-EPI should be defined at first mention and then used consistently thereafter. |
| Page 2 & Line No.26 | Kindly add comorbidities data in the manuscript, it can be summarized in tabular form for clear picturisation of result. |
| **Methods** |  |
| Page 4 & & Line No. 26-28 | Kindly reframe this sentence as meaning is not clearly inferenced from it. |
| Page 4-5 & & Line No. 31- 02 | Kindly remove this sentence as it is irrelevant to the context of this study. |
| **Discussion** |  |
| Page 8 & Line No. 29-30 | Kindly remove this line as it is irrelevant to context. |
| Page 9 & Line No. 05 | Kindly replace term “Overweight men” with “Overweight patients”. |
| Page 11 & Line No. 29- 30 | Kindly remove this sentence, as this can not be represented as limitation of the study. |
| **Tables** |  |
| Page 15-16 | Kindly arrange variable (Obesity and GFR category) of table 1 in accordance to correct scientific format |
| Page 16-17 | Kindly rewrite Table 2 in proper uniform format.  What “e” signifies please mention it in note section. |
| **Summary**:   - The data of co-morbidities in the manuscript should be clearly stated, they can be summarized in tabular form for a clear representation of the result. - Table 1 and 2 are suggested to be reframed in accordance to proper uniform format. | |

After reviewing this manuscript, it seems that flow and clarity of manuscript is average and needs revision in table 1 and 2 before acceptance.

1. Appropriateness of the Subject matter – average

2. Methods used - adequate

3 Information Contained - Valuable

4 Clarity of Presentation – Not Good

5 Statistical analysis - adequate

6 Conclusions – supported objective

7 Language – Average

8 Overall Quality - average

9 If acceptable, is it possible to reduce text? - Yes

10 Ethical Clearance – required
