# Supplementary material for: Dosage individualization proposed for anti-gout medications among the patients with gout
Source: PLoS One. 2021 Sep 17;16(9):e0257082. doi: 10.1371/journal.pone.0257082 (PMC8448378; doi:10.1371/journal.pone.0257082)
Supplement: Attachment — Submitted filename: Response to the reviewers and the editors comments_R4.docx [file pone.0257082.s006.docx]

**Reviewers' comments:**

**Reviewer's Responses to Questions**

**Comments to the Author**

1. If the authors have adequately addressed your comments raised in a previous round of review and you feel that this manuscript is now acceptable for publication, you may indicate that here to bypass the “Comments to the Author” section, enter your conflict of interest statement in the “Confidential to Editor” section, and submit your "Accept" recommendation.

**Reviewer #3: All comments have been addressed**

**Response:** Thank you very much for your confirmation.

**2. Is the manuscript technically sound, and do the data support the conclusions?**
The manuscript must describe a technically sound piece of scientific research with data that supports the conclusions. Experiments must have been conducted rigorously, with appropriate controls, replication, and sample sizes. The conclusions must be drawn appropriately based on the data presented.

**Reviewer #3: Yes**

**Response:** Thank you very much for your confirmation.

**3. Has the statistical analysis been performed appropriately and rigorously?**

**Reviewer #3: Yes**

**Response:** Thank you very much for your confirmation.

**4. Have the authors made all data underlying the findings in their manuscript fully available?**
The [PLOS Data policy](http://www.plosone.org/static/policies.action#sharing) requires authors to make all data underlying the findings described in their manuscript fully available without restriction, with rare exception (please refer to the Data Availability Statement in the manuscript PDF file). The data should be provided as part of the manuscript or its supporting information, or deposited to a public repository. For example, in addition to summary statistics, the data points behind means, medians and variance measures should be available. If there are restrictions on publicly sharing data—e.g. participant privacy or use of data from a third party—those must be specified.

**Reviewer #3: No**

**Response:** Full data would be made available upon request to the corresponding author. Data from individual patient would make the journal's space unnecessary space and we think these will not be of interest and importance to all the prospective researchers. The required information has already been supplied in S1 and S2 appendices.

**5. Is the manuscript presented in an intelligible fashion and written in standard English?**
PLOS ONE does not copyedit accepted manuscripts, so the language in submitted articles must be clear, correct, and unambiguous. Any typographical or grammatical errors should be corrected at revision, so please note any specific errors here.

**Reviewer #3: Yes**

**Response:** Thank you very much for your confirmation.

**6. Review Comments to the Author**

Please use the space provided to explain your answers to the questions above. You may also include additional comments for the author, including concerns about dual publication, research ethics, or publication ethics. (Please upload your review as an attachment if it exceeds 20,000 characters)

**Reviewer #3: The data of co-morbidities in the manuscript should be clearly stated, they can be summarized in tabular form for a clear representation of the result. Table 1 and 2 are suggested to be reframed in accordance to the correct scientific format.**

**Response:** The data of co-morbidities were already displayed at the 'S1 Appendix: Comorbidities of the patients'. Table 1 and 2, and S1 and S2 appendices have now been reframed in accordance to the correct scientific format.
